# Detecting Internal Defects in FRP-Reinforced Concrete Structures through the Integration of Infrared Thermography and Deep Learning

**DOI:** 10.3390/ma17133350

**Published:** 2024-07-06

**Authors:** Pengfei Pan, Rongpeng Zhang, Yi Zhang, Hongbo Li

**Affiliations:** 1 Xinhua College, Ningxia University, Yinchuan 750021, China; panpengfei1@stu.nxu.edu.cn; 2 College of Civil and Hydraulic Engineering, Ningxia University, Yinchuan 750021, China; 3 State Key Laboratory of Robotics, Shenyang Institute of Automation, Chinese Academy of Sciences, Shenyang 110016, China; zhangrongpeng@sia.cn; 4 School of Civil Engineering, Tsinghua University, Beijing 100086, China

**Keywords:** FRP, interfacial damage detection, Mask RCNN, infrared thermography

## Abstract

This study represents a significant advancement in structural health monitoring by integrating infrared thermography (IRT) with cutting-edge deep learning techniques, specifically through the use of the Mask R-CNN neural network. This approach targets the precise detection and segmentation of hidden defects within the interfacial layers of Fiber-Reinforced Polymer (FRP)-reinforced concrete structures. Employing a dual RGB and thermal camera setup, we captured and meticulously aligned image data, which were then annotated for semantic segmentation to train the deep learning model. The fusion of the RGB and thermal imaging significantly enhanced the model’s capabilities, achieving an average accuracy of 96.28% across a 5-fold cross-validation. The model demonstrated robust performance, consistently identifying true negatives with an average specificity of 96.78% and maintaining high precision at 96.42% in accurately delineating damaged areas. It also showed a high recall rate of 96.91%, effectively recognizing almost all actual cases of damage, which is crucial for the maintenance of structural integrity. The balanced precision and recall culminated in an average *F*1*-score* of 96.78%, highlighting the model’s effectiveness in comprehensive damage assessment. Overall, this synergistic approach of combining IRT and deep learning provides a powerful tool for the automated inspection and preservation of critical infrastructure components.

## 1. Introduction

In the realm of modern civil engineering, the utilization of Fiber-Reinforced Polymer (FRP) to reinforce concrete structures has become a hallmark of innovation, significantly augmenting their strength, durability, and resistance to corrosion [1,2,3]. This method of reinforcement not only boosts the load-bearing capabilities of these structures but also contributes to their longevity, playing a pivotal role in enhancing the resilience of infrastructure against disasters [4,5]. Despite these advancements, the existence of invisible defects within the interfacial layer of FRP-reinforced concrete structures—such as micro-cracks, delamination, or voids—represents a profound challenge [6]. These hidden flaws undermine the structural integrity and stability, potentially leading to the progressive deterioration of the construction and jeopardizing its overall safety [7,8,9].

Traditionally, the detection of such defects has relied on methods like visual inspections, acoustic testing, and electromagnetic detection techniques [10,11,12]. While these approaches have their merits, they fall short in identifying invisible damage within the complex matrix of FRP-reinforced concrete structures, particularly those that are deeply embedded [13,14]. The evolution of image processing technologies has introduced new avenues for identifying these hidden damages. Nevertheless, conventional RGB image processing techniques often suffer from diminished effectiveness due to lighting condition variances, which can obscure the detection of deep-seated defects [15].

Addressing these technological gaps, our study introduces a groundbreaking methodology that marries infrared thermography with advanced deep learning models, specifically employing the Mask R-CNN framework, for the automated detection and segmentation of hidden defects [16,17,18]. Infrared thermography, capable of detecting minute variations in temperature distribution unaffected by lighting conditions, serves as an ideal indicator of underlying structural issues [19]. By harnessing the power of thermal imaging data within deep learning models, we unlock the potential for automatic identification and detailed segmentation of structural damages, transcending the limitations of conventional detection methods.

Infrared thermography, a non-destructive testing method, has been increasingly applied in several scientific sectors, among which are mechanical engineering [20] and energy saving [21]. The temperature readings are presented visually at the end of the inspection process [22,23,24]. Unlike visible light, IR has longer wavelengths that elude human vision [25,26,27]. IRT stands out in non-destructive evaluation (NDE) for detecting structural anomalies unseen by conventional means, as affirmed by extensive research. In large-scale civil structures, passive IRT is especially useful for NDE purposes, utilizing solar radiation as a heat source when artificial heating is impractical [25,28]. Studies suggest that the timing of image capture is crucial to maximize thermal contrast, and daily thermal variations can be sufficient for IRT to discern defects even in shaded areas [29,30,31]. The duality of visible RGB and thermal imaging is associated with a combination of advantages and limitations. Visible imaging offers high resolution and cost-effectiveness but can falter with lighting variations [32,33,34]. Thermal imaging, while less affected by light, reveals subsurface anomalies but at the expense of resolution and clarity [35,36]. Modern developments have seen manufacturers designing cameras that concurrently capture both visible and thermal data, enriching the contextual details of thermal diagnostics [37].

Our research delineates the novel integration of infrared thermography and the Mask R-CNN deep learning algorithm to revolutionize the detection and categorization of internal defects. This integration not only amplifies the precision and speed of defect identification but also ensures consistent performance across varied environmental conditions. Through this pioneering approach, we aim to significantly advance the field of structural health monitoring, setting a new benchmark for the automated and accurate assessment of infrastructure integrity.

## 2. Methodology

**Figure 1:**
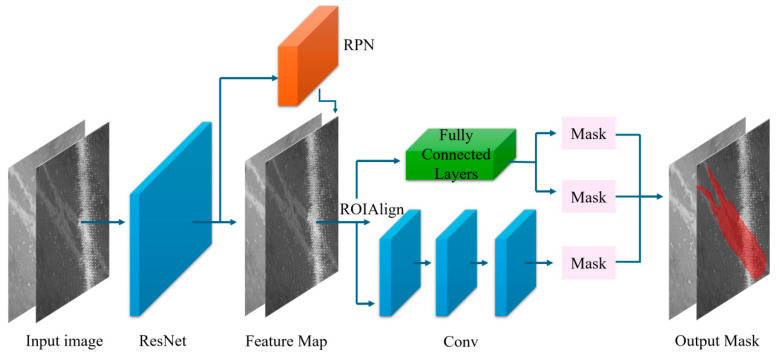
Proposed framework.

The Mask R-CNN [16] framework is structured around three core components: the backbone, the Region Proposal Network (RPN), and RoIAlign, illustrated in Figure 1. The architecture integrates a Feature Pyramid Network (FPN)-styled backbone designed to extract hierarchical image features [38,39,40]. The backbone predominantly comprises ResNet architectures, specifically ResNet41, ResNet50, ResNet65, and ResNet101 [41]. The configuration of ResNet50 includes 48 convolutional layers, complemented by one MaxPool and one Average Pool layer, supporting approximately 3.8 × 10^9^ floating point operations.

### 2.1. Sample Preparation and Damage Induction

This research involves systematically introducing defects into reinforced concrete (RC) components, such as RC beams and slabs, to replicate invisible interface defects in CFRP (Carbon Fiber-Reinforced Polymer)-reinforced structures. Initially, the RC components undergo controlled procedures to create micro-cracks, simulating common damage observed in aging concrete infrastructure. Subsequently, two layers of CFRP sheets are applied externally to these pre-damaged components using an industry-standard epoxy resin. This method not only replicates the practical application of BFRP in reinforcing concrete structures but also facilitates the examination of how CFRP interacts with existing defects. Such studies are essential to assess the real-world efficacy and potential limitations of CFRP as a strengthening solution in structural engineering.

### 2.2. Thermal Treatment and Imaging

Following the damage induction, the specimens undergo a thermal treatment process. A spotlight heating technique is employed to uniformly heat the surface of the FRP-reinforced concrete samples. Given the higher specific heat capacity of air compared to the surrounding materials, the areas with defects, filled with air, retain more heat. This differential in heat retention makes the damaged areas more discernible in thermal images. The spotlight’s heat causes these defects to exhibit higher temperatures than the intact portions of the concrete, facilitating their identification through subsequent thermal imaging.

### 2.3. Thermal Imaging Acquisition

Thermal images of the heated samples are captured using a commercial high-resolution infrared camera. This camera features a resolution of 640 × 480 pixels, thermal sensitivity of <0.04 °C at +30 °C, a temperature range of −40 °C to +500 °C, an accuracy of ±2 °C or ±2% of the reading, and a spectral range of 7.5–14 µm. These images serve as the primary data source for identifying the locations and extents of the internal defects. The camera is calibrated to accurately detect temperature variations, ensuring that even subtle differences indicative of damage are recorded.

Image fusion necessitates precise alignment between the individual images. As per Rao et al. [42], a normalized cross-correlation (NCC) technique is effective for matching images with distinct structural content. In this method, the reference image remains stationary while the comparative image shifts incrementally, pixel by pixel. The correspondence between the images at each shift is evaluated by a correlation coefficient, with values ranging from 0 to 1, where 1 denotes a perfect match. The optimal alignment is found at the position with the highest correlation coefficient.

**Figure 2:**
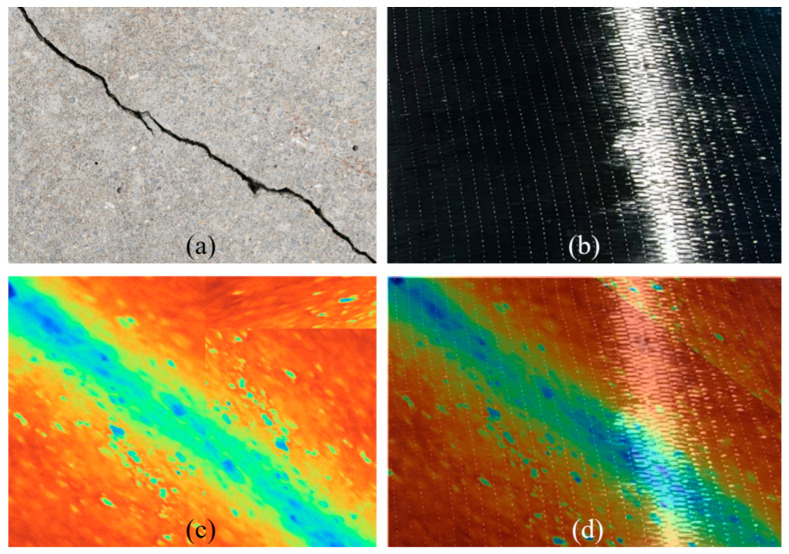
(**a**) Unbonded damage image; (**b**) bonded damage image; (**c**) bonded thermal image; and (**d**) fusion image.

As depicted in Figure 2, the original resolution of thermal images is usually lower than that of the photographs capturing invisible damage at the FRP–concrete interface; hence, the thermal image requires upscaling to match the visible image’s details. Post-scaling, the NCC technique is employed to find the maximum correlation coefficient location, adjusting the thermal image to the visible image’s scale through zero-padding. A qualitative assessment of the alignment accuracy is performed by merging the images. Figure 2 illustrates (a) the concrete surface with induced damage, (b) the FRP–concrete interface damage photo, (c) the zero-padded thermal image, and (d) the blended image of the FRP–concrete damage photo with the padded thermal image, showcasing an exemplary scenario. This alignment method is systematically applied across all image pairs in the dataset, utilizing various color palettes, such as the iron palette for the thermal images.

### 2.4. Deep Learning Model for Object Segmentation

The core of our methodology lies in leveraging advanced deep learning for automated damage detection and segmentation, specifically utilizing the Mask R-CNN model. We input captured thermal images into this model, which is adapted from a traditional ResNet architecture and optimized for semantic segmentation tasks. This enhanced Mask R-CNN model is meticulously trained to identify and precisely segment the thermal signatures indicative of damage within the images. The segmentation process effectively isolates the damaged areas, allowing for the accurate localization of defects and facilitating a detailed quantitative assessment of internal deficiencies.

The training phase of our deep learning model is comprehensive, utilizing a dataset that includes thermal images of various FRP-reinforced concrete specimens, both damaged and undamaged. This phase employs a robust combination of supervised learning techniques. The Mask R-CNN model undergoes iterative refinements through a feedback loop that focuses on enhancing the accuracy of its damage detection and segmentation capabilities. We validate the effectiveness of the model using a separate set of thermal images not previously seen during the training phase, thereby ensuring the model’s robustness and reliability in consistently detecting and segmenting internal defects across diverse conditions. This methodical approach ensures that the model not only learns from the data but also generalizes well to new, unseen scenarios, making it a powerful tool for structural health monitoring.

## 3. Experimental Study

### 3.1. Preparation of Test Specimens

**Figure 3:**
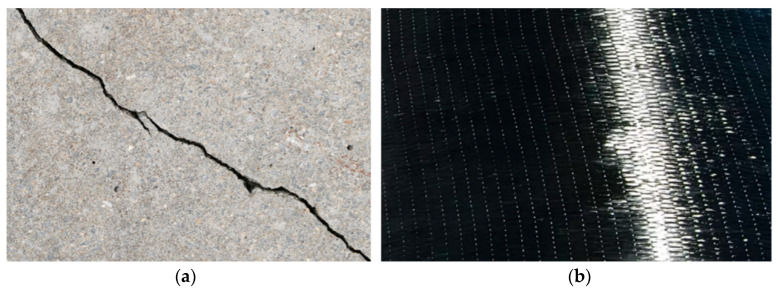
Specimens: (**a**) unbonded concrete and (**b**) bonded concrete.

This research begins with systematically creating defects in RC components, specifically RC beams, to simulate invisible defects at the interface of CFRP-reinforced concrete structures. Initially, the RC components underwent controlled cracking procedures to introduce micro-cracks, closely resembling typical damage in aging concrete infrastructures. After creating these cracks, CFRP sheets were externally bonded to the damaged RC components using industry-standard epoxy resin, as illustrated in Figure 3. This critical step mimics the real-world application of CFRP for strengthening concrete structures while introducing interface defects invisible to the naked eye. Precisely placing CFRP over these pre-damaged areas allows for studying how such reinforcements interact with existing defects, which is crucial for understanding the effectiveness and limitations of CFRP reinforcement in practical scenarios.

### 3.2. Setup Equipment

The experimental setup included a thermal imaging camera and a spotlight for heating the specimens. The thermal camera was selected based on its high resolution and sensitivity to temperature variations, crucial for detecting subtle changes in the thermal profile indicative of underlying defects. The spotlight, capable of emitting a consistent and controlled heat output, was used to uniformly heat the surface of each slab. Each concrete slab was subjected to a precise heating protocol to enhance the visibility of defects in thermal images. The slabs were heated for 10 min to ensure adequate heat penetration and retention within the defects. This duration was determined based on preliminary trials that optimized the visibility of defects while avoiding any potential damage to the FRP material or the concrete.

## 4. Results and Discussion

### 4.1. Database

**Figure 4:**
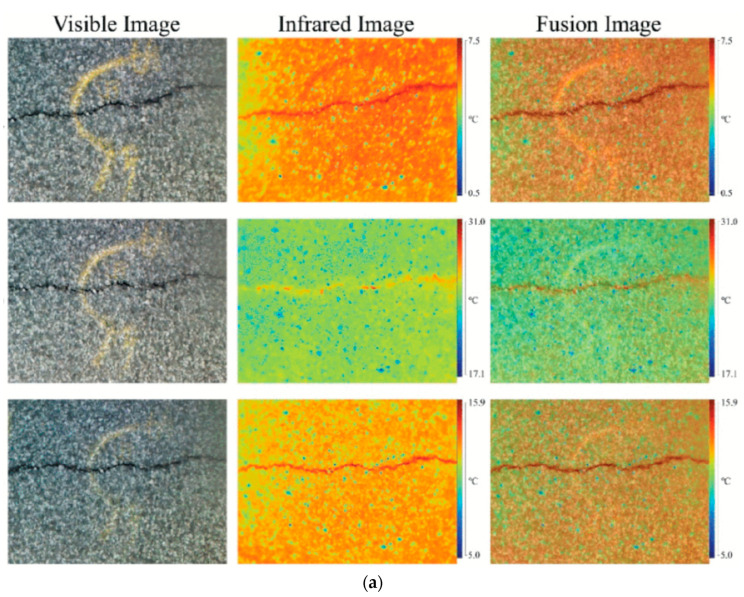

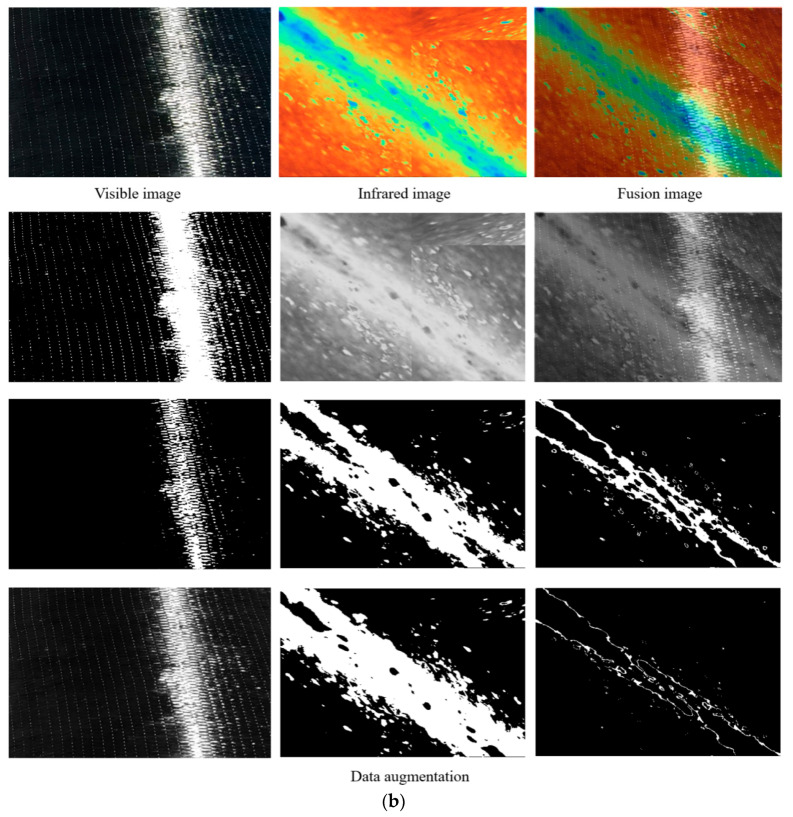
Database used in this study. (**a**) Open-source dataset [43]. (**b**) Dataset of this study.

In this study, we identified an open-source dataset to address the scarcity of open benchmark datasets for crack detection using IRT. A previous study [43] established an enhanced open benchmark dataset specifically designed for IRT-based crack detection. This dataset comprises four types of images: visible images, infrared images, fusion images combining both visible and infrared data, and ground truth images for accurate evaluation and training purposes, as shown in Figure 4a. By combining diverse data sources, we significantly increased the variability and volume of our dataset, which is crucial for developing more effective and generalized models for IRT-based crack detection. This mixed dataset approach not only strengthens our training process but also helps in validating the predictive capabilities of our models under varied conditions.

For our dataset, given its relatively limited size, we employed data augmentation techniques to enhance its utility and robustness for training purposes. This involved artificially expanding the diversity of the dataset through several image processing techniques. Specifically, we introduced noise to the images to simulate real-world irregularities and applied binarization to emphasize the contrast, making the crack features more pronounced and easier for the deep learning models to identify and learn from. These data augmentation methods help mitigate the risk of overfitting by providing a broader array of training examples and thus improve the model’s ability to generalize across different and unseen data scenarios in crack detection using IRT, as shown in Figure 4b.

### 4.2. Model Evaluation

The model’s loss function integrates several components to optimize performance. Specifically, the classification loss and the bounding box regression loss are combined. The classification loss (*L_cls_*) is computed using binary cross-entropy, represented as

(1) $$L_{cls}=-\frac{1}{N_{cls}}\sum_{i=1}^{N_{cls}}\left(y_{i}\log\left(p_{i}\right)+\left(1-y_{i}\right)\log\left(1-p_{i}\right)\right)$$

where *p_i_* is the predicted probability that the anchor *i* is positive, *y_i_* is the ground truth label (1 if the anchor is positive, 0 otherwise), and *N_cls_* is the size of the mini-batch.

For the bounding box regression, the Smooth *L*_1_ loss is utilized, which is less sensitive to outliers compared to other loss functions. This is expressed as

(2) $$L_{bbox}=\frac{1}{N_{bbox}}\sum_{i=1}^{N_{bbox}}smooth_{L1}\left(t_{i}-{\overset{⌢}{t}}_{i}\right)$$

where *t_i_* denotes the predicted coordinates of the bounding box, ${\overset{⌢}{t}}_{i}$ is the ground truth coordinates for the positive anchor, and *N_bbx_* represents the number of anchor locations.

The experiments on the deep learning model were performed to detect invisible damage in the thermal images. The Mask R-CNN was trained for 100 epochs. The accuracy, specificity, precision, and recall were compared for the different methods as well as for the method used in this study. The trained neural network was used to classify the thermal images. The parameters used to evaluate the performance are

(3) $$Accuracy=\frac{TP+TN}{TP+FP+TN+FN}$$

(4) Recall=TPTP+FN

(5) $$\mathrm{Pr}ecision=\frac{TP}{TP+FP}$$

(6) F1-score=2×Precision×RecallPrecision+Recall

(7) $$Specificity=\frac{TN}{TN+FP}$$

The metrics *TP* (true positive), *TN* (true negative), *FP* (false positive), and *FN* (false negative) are fundamental indicators used to measure the performance of the proposed models. The evaluation metrics such as the accuracy, specificity, recall, precision, and *F*1-*score* are summarized in Table 1. These values assess how effectively the deep learning model identifies invisible damage in thermal images. In the overview of model efficacy, ResNet50 emerges as the most accurate, boasting a 94.75% accuracy rate, which indicates its superior ability in making correct predictions. When it comes to specificity, ResNet101 leads the group with a rate of 90.95%, reflecting its proficiency in identifying instances where no damage is present. ResNet50 continues to excel with the highest precision of 95.60%, suggesting its predictions of damage are reliably accurate. Meanwhile, ResNet101 outperforms others in recall with a rate of 89.80%, showcasing its effectiveness in capturing all instances of actual damage within the dataset. The *F*1*-score*, a crucial indicator of a model’s balanced performance in precision and recall, is again topped by ResNet50, with an *F*1*-score* of 93.45%, indicating its consistent and reliable detection capability with minimal false identifications. These subscripted figures hint at further clarifications provided in the study, with ResNet50 generally demonstrating the best performance across multiple metrics, thus potentially offering a more refined tool for identifying structural integrity in civil engineering applications.

**Figure 5:**
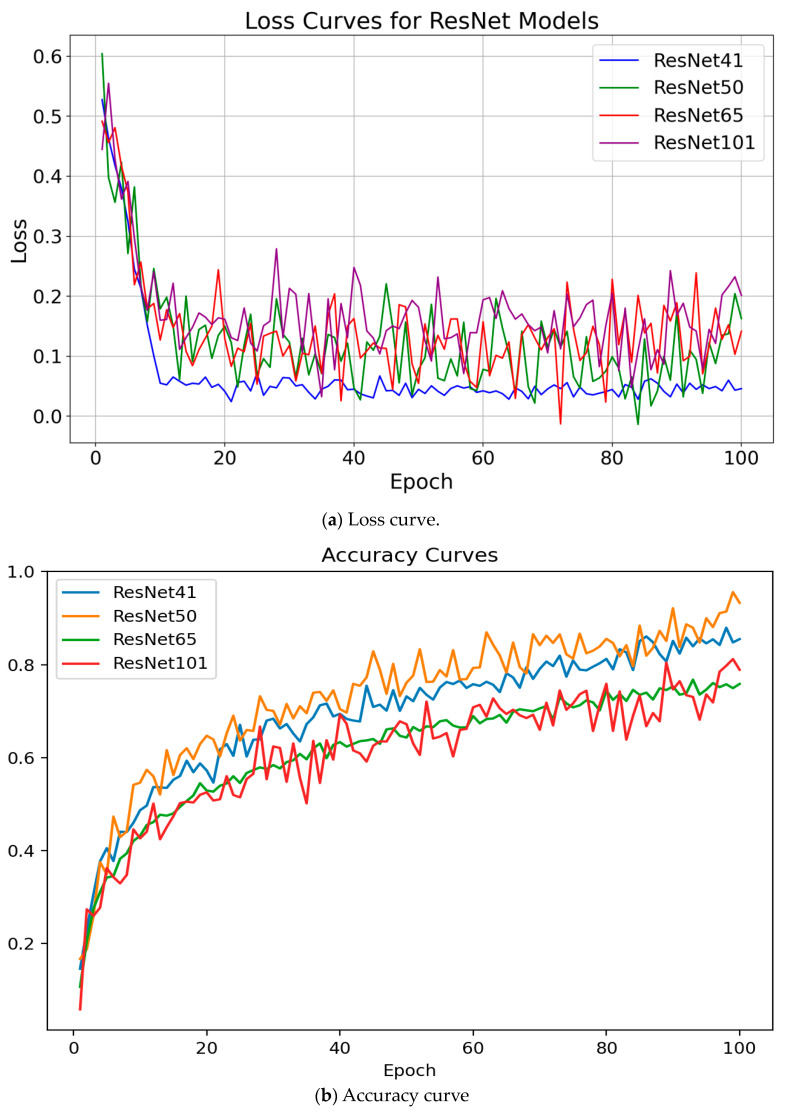
Loss curve and accuracy curve.

Figure 5a illustrates the distinct behavior of loss curves corresponding to infrared and fused images when subjected to segmentation modeling. We observed a general trend toward convergence in loss across the majority of segmentation models as epochs increased. Figure 5b presents the accuracy curve for the segmentation models over the training epochs. When examining other accuracy metrics, fluctuations were observed in the models. Despite these variations, the general trend indicated that the segmentation models tended to reach stability in accuracy measurements as the epochs progressed.

### 4.3. Cross Validation

To rigorously assess the Mask R-CNN model’s capability in detecting invisible damage at the FRP–concrete interface using thermal images, a 5-fold cross-validation strategy was employed. Each fold served sequentially as the testing set while the remainder of the data constituted the training set. This approach ensured a comprehensive evaluation, treating the task as a binary classification problem and distinguishing between damaged and undamaged areas. Among the various architectures tested, the ResNet50 configuration emerged as the most effective. For each fold, a Confusion Matrix (CM) was generated, facilitating a nuanced understanding of the model’s classification prowess. The performance in the metrics across all folds, including their aggregated means, are presented in Table 2. The ResNet50 model achieved mean accuracy, specificity, precision, recall, and *F*1*-score* values of 96.98%, 97.36%, 96.60%, 97.32%, and 96.93%, respectively.

## 5. Conclusions

In conclusion, our study aimed to determine the benefits of integrating RGB and thermal imaging for enhancing a deep learning model’s ability to detect structural damage within large-scale civil infrastructures. The image data, collected using a cost-effective dual RGB and thermal camera, underwent rigorous alignment and manual annotation for semantic segmentation. In concluding our research on the detection of invisible damage at the CFRP–concrete interface using a Mask R-CNN model and thermal imaging, the following key findings are highlighted:

(1) The implementation of the Mask R-CNN model yielded an impressive average accuracy of 96.28% over a 5-fold cross-validation, indicating a robust capability to identify invisible damage within the tested samples.
(2) The model demonstrated consistent performance in specificity and precision across all folds, averaging 96.78% and 96.42%, respectively. This consistency underscores the reliability of the model in correctly identifying true negatives and its precision in the delineation of damaged areas.
(3) With an average recall of 96.91%, the model effectively recognized almost all the actual cases of damage, a critical metric for ensuring that damage is not overlooked and that the safety of structures is not compromised.
(4) The balanced measure of the model’s precision and recall is reflected in an average *F*1*-score* of 96.78%, suggesting that the model maintains a harmonious balance between the accuracy of damage detection and the completeness of damage identification.

However, it is important to note that the findings of this study are based solely on tests conducted with CFRP materials. As such, while the results are promising, they should be interpreted with caution. Further testing with different types of FRP materials is necessary to fully validate the method’s effectiveness and ensure its broad applicability.

## Figures and Tables

**Table 1:** Comparison of performance by different models.

Method	Accuracy	Specificity	Precision	Recall	F1
ResNet41	90.15%	88.20%	92.35%	85.45%	88.90%
ResNet50	94.75%	93.00%	95.60%	91.30%	93.45%
ResNet65	92.40%	89.85%	93.25%	88.50%	90.75%
ResNet101	93.60%	90.95%	94.70%	89.80%	92.25%

**Table 2:** Comparison of the performance in the metrics for each fold.

Fold	Accuracy	Specificity	Precision	Recall	F1
Fold-1	95.55%	96.10%	97.00%	95.12%	96.55%
Fold-2	96.80%	98.00%	96.70%	98.77%	97.73%
Fold-3	97.70%	97.40%	99.00%	97.36%	98.17%
Fold-4	94.75%	95.50%	92.10%	96.82%	94.57%
Fold-5	96.60%	96.90%	97.30%	96.50%	96.89%
Mean	96.28%	96.78%	96.42%	96.91%	96.78%

## Data Availability

The dataset and corresponding algorithm used in this result will be published on GITHUB. Data will be made available on request.
